# Socioeconomic Disparities and the Role of Comorbidity in Hospital Mortality: A Dutch Nationwide Critical Care Cohort Study

**DOI:** 10.1097/CCM.0000000000007271

**Published:** 2026-07-21

**Authors:** Jip W.T.M. de Kok, Daniëlle J.M. Koornneef, Fabian Termorshuizen, Ronny M. Schnabel, Iwan C.C. van der Horst, Nicolette F. de Keizer, Sylvia Brinkman, Bas C.T. van Bussel

**Affiliations:** 1 Department of Intensive Care Medicine, Maastricht University Medical Centre+, Maastricht, the Netherlands.; 2 Cardiovascular Research Institute Maastricht (CARIM), Maastricht University, Maastricht, the Netherlands.; 3 National Intensive Care Evaluation (NICE) Foundation, Amsterdam, the Netherlands.; 4 Department of Medical Informatics, Amsterdam Public Health Institute, Amsterdam UMC, University of Amsterdam, the Netherlands.; 5 Albert Schweitzer Hospital, Dordrecht, the Netherlands.; 6 Care and Public Health Research Institute (CAPHRI), Maastricht University, Maastricht, the Netherlands.

**Keywords:** comorbidity, critical care, epidemiology, hospital mortality, public health, socioeconomic factors

## Abstract

**OBJECTIVES:**

Lower socioeconomic status (SES) is associated with a higher prevalence of comorbidities and mortality. The association between SES and mortality, as well as the effect-modifying role of specific comorbidities, are not yet fully understood within the critical care context of insurance-based universal healthcare systems. To inform health policy and practice, we examined socioeconomic disparities in Dutch ICU admissions, considering comorbidities in the context of hospital mortality.

**DESIGN:**

Nationwide retrospective cohort study.

**SETTING:**

All 79 Dutch ICUs were included.

**Patients:**

We used data from 588,568 ICU admissions (2014–2022) linked with household-level SES data.

**INTERVENTIONS:**

None.

**MEASUREMENTS AND MAIN RESULTS:**

We used mixed-effects logistic regression models to investigate the association between SES quintiles and hospital mortality. We adjusted models for age, sex, year of admission, acute physiologic derangement, and comorbidities. Also, we investigated the effect-modifying role of seven comorbidities: respiratory insufficiency, renal insufficiency, cancer, cardiovascular insufficiency, diabetes mellitus, cirrhosis, and immunological insufficiency. Unadjusted odds ratios (ORs) for hospital mortality were higher among patients with lower SES. After adjustment, SES quintiles remained significantly different from the middle quintile, with attenuated ORs (95% CI): 1.6 (1.5–1.6), 1.1 (1.0–1.1), 1.0 (0.9–1.0), and 0.9 (0.9–1.0), respectively. The association was most pronounced in the lowest SES quintile and persisted in patients with and without specific comorbidities.

**CONCLUSIONS:**

ICU patients with a lower SES face poorer survival prospects, irrespective of comorbidities. This highlights the persistent health disparities that may influence ICU outcomes. SES should be considered for interpreting outcome measures, designing healthcare policies, and targeting interventions to improve health equity.

**KEY POINTS**

**Question:** Is lower socioeconomic status associated with higher hospital mortality in Dutch intensive care patients, and do comorbidities have an impact on this relationship?

**Findings:** Hospital mortality was significantly higher among patients with a lower socioeconomic background irrespective of age, sex, and acute physiologic derangement. The association was most pronounced in the lowest socioeconomic group and persisted in patients with and without comorbidities.

**Meaning:** We identified a substantial health disparity in Dutch intensive care patients, highlighting the need for considering SES in designing healthcare policies and targeting interventions to improve health equity.

The most severely ill patients are treated in the ICU, resulting in a hospital mortality rate of approximately 13% in Dutch ICUs (1). Consequently, risk factors including demographics, comorbidities, and laboratory assessments measuring physiologic derangement have been considered as predictors of mortality (2). Nevertheless, socioeconomic status (SES), defined as an individual’s relative social position in society based on employment, wealth, and education (3–5), is largely overlooked when assessing ICU performance using standardized, case-mix-adjusted outcome measures. Lower SES is consistently linked to a higher incidence of adverse health outcomes, a poorer health trajectory, and increased healthcare utilization (3–9). This phenomenon, known as the “social gradient,” is recognized as a primary public health challenge (3–5), also in the ICU (10–12).

Previous studies across several countries and healthcare systems have examined the association between SES and mortality in ICU patients. While some report higher mortality among patients with lower SES (12–17), others show no statistically significant association (18–26). This observed heterogeneity is likely due to variations in healthcare systems (i.e., nonuniversal [24, 27–29], tax-funded universal [13–17, 30], and insurance-based universal [19–22, 31] healthcare systems) and, importantly, differences in SES conceptualization and measurement. Many studies rely on area-based indices (14–16, 18, 23–26, 30, 32–34), which introduce misclassification of individual circumstances, particularly in settings with pronounced within-area socioeconomic variation. Conversely, studies with individual-based indices (often survey-based) (13, 19–22, 31, 35) present smaller sample sizes and are prone to selection bias due to nonresponse (36). Furthermore, adjustments for comorbidities were commonly done using composite indices, instead of individual comorbidities (14, 15, 17, 18, 22–25, 30, 31, 33, 37), while each comorbidity could differentially modify the SES–mortality association.

Therefore, the present study uses a nationwide ICU cohort that links household-level SES to individual comorbidities within a universally covered insurance-based healthcare system. We formulated three hypotheses: 1) lower SES is associated with higher hospital mortality; 2) this association is independent of age, sex, acute physiologic derangements during the first 24 hours, and comorbidities; and 3) the association differs between patients with and without preexisting comorbidities. This approach aims to offer a nuanced understanding of the social gradient in critical care outcomes to inform targeted policies and promote health equity.

## MATERIALS AND METHODS

This cohort study used data from the National Intensive Care Evaluation (NICE) registry (38) and included patient data from all Dutch ICUs from January 1, 2014, to December 31, 2022. The dataset contained demographic, physiologic, laboratory, ICU, and hospital mortality data of all consecutive patients admitted to all 79 Dutch ICUs (39). The Acute Physiology and Chronic Health Evaluation III Acute Physiology Score (APS) was used to quantify acute physiologic derangement in patients within the first 24 hours in the ICU (40). Seven comorbidities (medical conditions besides primary ICU diagnoses present before hospitalization) were considered: respiratory insufficiency, renal insufficiency, cancer, cardiovascular insufficiency, diabetes mellitus, cirrhosis, and immunological insufficiency. The NICE data were deterministically linked to household-level SES data from the National Database of Statistics Netherlands. The SES score is based on component scores for wealth, education level, and employment history and was divided into quintiles based on the entire study population. Details on data extraction, linkage, inclusion, and preparation are provided in the **Supplementary Methods** (https://links.lww.com/CCM/I25) section and have been comprehensively described elsewhere (41).

### Mixed-Effects Logistic Regression

We used mixed-effects logistic regression models to analyze the association between SES quintiles and hospital mortality by employing generalized linear mixed-effects models with a logit link function for the binomial distribution of the response variable. The models incorporated normally distributed random intercepts at the logit scale for ICUs and used the Nelder-Mead optimization algorithm, resulting in good convergence.

First, we investigated an unadjusted model (model 1) to analyze SES quintiles as categorical determinants of hospital mortality, with the middle quintile (quintile 3) as reference to provide effect estimates for lower and higher SES quintiles. Second, model 1 was additionally adjusted for age, sex, and year of admission (model 2). Third, model 2 was extended by individually adding each comorbidity separately, including an interaction term between the specific comorbidity and SES (models 3a–3g). The results of these models were then stratified by the presence or absence of each comorbidity to assess whether the association between SES quintiles and mortality differed by comorbidity status (models 3a–3g, present vs. absent). Finally, to obtain a fully adjusted model, model 2 was additionally adjusted for all investigated comorbidities simultaneously (without interaction terms) and APS (model 4). An overview of these models is presented in **Table S1** (https://links.lww.com/CCM/I25). A sensitivity analysis was included by adjusting model 2 for the number of comorbidities (0, 1, 2, or > 2) with an interaction with SES. We also conducted two extreme-case sensitivity analyses in which patients with unlinked or missing SES were assigned the minimum or, alternatively, the maximum SES value to assess potential bias from missingness. Wald tests and likelihood ratio tests were performed on models 3a–3g to assess the overall joint effect of SES within each comorbidity stratum. Wald tests were performed on each of the models to assess the overall joint effect of SES in general. Since APS could influence the associations between comorbidities and hospital mortality, model 2, without APS, was used to investigate the role of comorbidities. To improve convergence, age and APS were standardized to a *z*-score. For all models, the odds ratios (ORs) of the fixed effects were calculated, with 95% CIs. ORs stratified by the presence or absence of comorbidity were calculated by summing relevant coefficients for SES and interaction effects from models 3a–3g. CIs were based on the se of the combined estimate. The resulting estimates for the differences in log-odds and associated 95% CIs were exponentiated to obtain the ORs with 95% CIs.

We focused on effect sizes presented as ORs with 95% CI rather than *p* values, which provide little added value in the present study, particularly given the large sample size (42, 43). We did not correct for multiple testing in the comorbidity-specific interaction analyses to avoid inflating type II errors; *p* values should be interpreted accordingly (44, 45). Results are based on calculations by the authors in project number 9247 using nonpublic microdata from Statistics Netherlands. Analyses were performed using R Version 4.4.0 (R Core Team, Vienna, Austria). We received a waiver of informed consent for the study protocol entitled “Observationeel onderzoek met patiëntgebonden gegevens routinematig verzameld bij IC’s in Nederland voor de Nationale Intensive Care Evaluatie (NICE) kwaliteitsregistratie” as adjudicated by the Medical Review Ethics Committee of Amsterdam University Medical Centre, approved on September 6, 2023. The study was performed in accordance with the Helsinki Declaration of 1975 and applicable national regulations [reference number: 2023.0259]. We followed the Strengthening the Reporting of Observational Studies in Epidemiology and Reporting of studies Conducted using Observational Routinely collected Data guidelines.

## RESULTS

In summary, from January 1, 2014, to December 31, 2022, 703,773 ICU records were collected, of which 79,935 were ineligible for analysis based on the exclusion criteria. Of the eligible ICU records, 18,082 (2.9%) records could not be linked by Statistics Netherlands, and 17,188 (2.8%) had no available SES data within the two years preceding admission (**Table S2**, https://links.lww.com/CCM/I25). The final study population consisted of 588,568 ICU records with SES scores (41).

SES quintiles showed differences in severity of illness, comorbidities, mortality, and demographics (**Table 1**). The low and average SES quintiles included older patients on average (68.8 and 68.4 years), and the lowest SES quintile contained fewer men (53.3%) than the other quintiles. Compared to the highest and high SES quintiles, the lowest and low quintiles had higher APS (48.0 and 46.3 vs. 42.7 and 41.0). Comorbidities were unevenly distributed across the SES quintiles. The prevalence of respiratory insufficiency, renal insufficiency, and diabetes mellitus was higher in the lowest and low SES quintiles, whereas cancer was less common in the lowest SES quintile than in the high and highest quintiles. The prevalence of immunological insufficiency was relatively consistent across SES quintiles.

**TABLE 1.** Descriptive Statistics of the Socioeconomic Status Quintiles in Dutch ICU Patients. Data are Presented as Mean ± sd or Numbers and Percentages. Socioeconomic Status Categories Were Based on Quintiles, Ranging From the Lowest to the Highest Socioeconomic Status

Variables	Lowest SES (n = 118,476)	Low SES (*n* = 122,175)	Medium SES (*n* = 119,076)	High SES (*n* = 112,099)	Highest SES (*n* = 116,742)	Total Cohort (*n* = 588,568)
Age, mean (sd)	61.1 (16.8)	68.8 (13.6)	68.4 (12.7)	61.5 (15.2)	57.4 (14.7)	63.5 (15.3)
Sex (male), *n* (%)	63,086 (53.2)	71,369 (58.4)	75,228 (63.2)	71,776 (64.0)	77,388 (66.3)	358,847 (61.0)
Acute Physiology Score, mean (sd)	48.0 (27.6)	46.3 (25.7)	45.0 (25.2)	42.7 (24.9)	41.0 (24.6)	44.6 (25.7)
Respiratory insufficiency, *n* (%)	24,278 (20.5)	23,072 (18.9)	18,729 (15.7)	13,710 (12.2)	10,211 (8.7)	90,000 (15.3)
Renal insufficiency, *n* (%)	8,817 (7.4)	8,620 (7.1)	7,222 (6.1)	5,027 (4.5)	3,754 (3.2)	33,440 (5.7)
Cancer, *n* (%)	5,642 (4.8)	7,226 (5.9)	7,858 (6.6)	7,200 (6.4)	8,079 (6.9)	36,005 (6.1)
Cardiovascular insufficiency, *n* (%)	5,579 (4.7)	6,394 (5.2)	5,773 (4.8)	4,430 (4.0)	3,874 (3.3)	26,050 (4.4)
Diabetes mellitus, *n* (%)	26,186 (22.1)	25,570 (20.9)	21,303 (17.9)	16,231 (14.5)	12,512 (10.7)	101,802 (17.3)
Cirrhosis, *n* (%)	2,643 (2.2)	1,421 (1.2)	1,315 (1.1)	1,246 (1.1)	1,290 (1.1)	7,915 (1.3)
Immunological insufficiency, *n* (%)	9,675 (8.2)	10,201 (8.3)	10,520 (8.8)	9,614 (8.6)	10,012 (8.6)	50,022 (8.5)
Hospital mortality, *n* (%)	19,932 (16.8)	16,805 (13.8)	14,720 (12.4)	10,763 (9.6)	9,259 (7.9)	71,479 (12.1)

SES = socioeconomic status.

In the unadjusted model (**Fig. 1**, model 1), lowest and low SES showed higher ORs (95% CI) of 1.4 (1.4–1.4) and 1.1 (1.1–1.1) compared with the reference middle SES (quintile 3), while the highest and high SES quintiles showed lower ORs of 0.6 (0.6–0.6) and 0.8 (0.7–0.8), respectively. These effects persisted after adjusting for potential confounders (Fig. 1, models 2 and 4). Although the strength of the ORs for most quintiles decreased, the OR for the lowest SES quintile increased after adjustment. The fully adjusted model, including age, sex, year of admission, all comorbidities, and APS (Fig. 1, model 4), showed, compared with the middle quintile (reference), ORs (95% CI) of 1.6 (1.5–1.6), 1.1 (1.0–1.1), 1.0 (0.9–1.0), and 0.9 (0.9–1.0) for the lowest, low, high, and highest SES quintiles, respectively. Please refer to **Tables S3**–**S13** (https://links.lww.com/CCM/I25) for more details.

**Figure 1.**
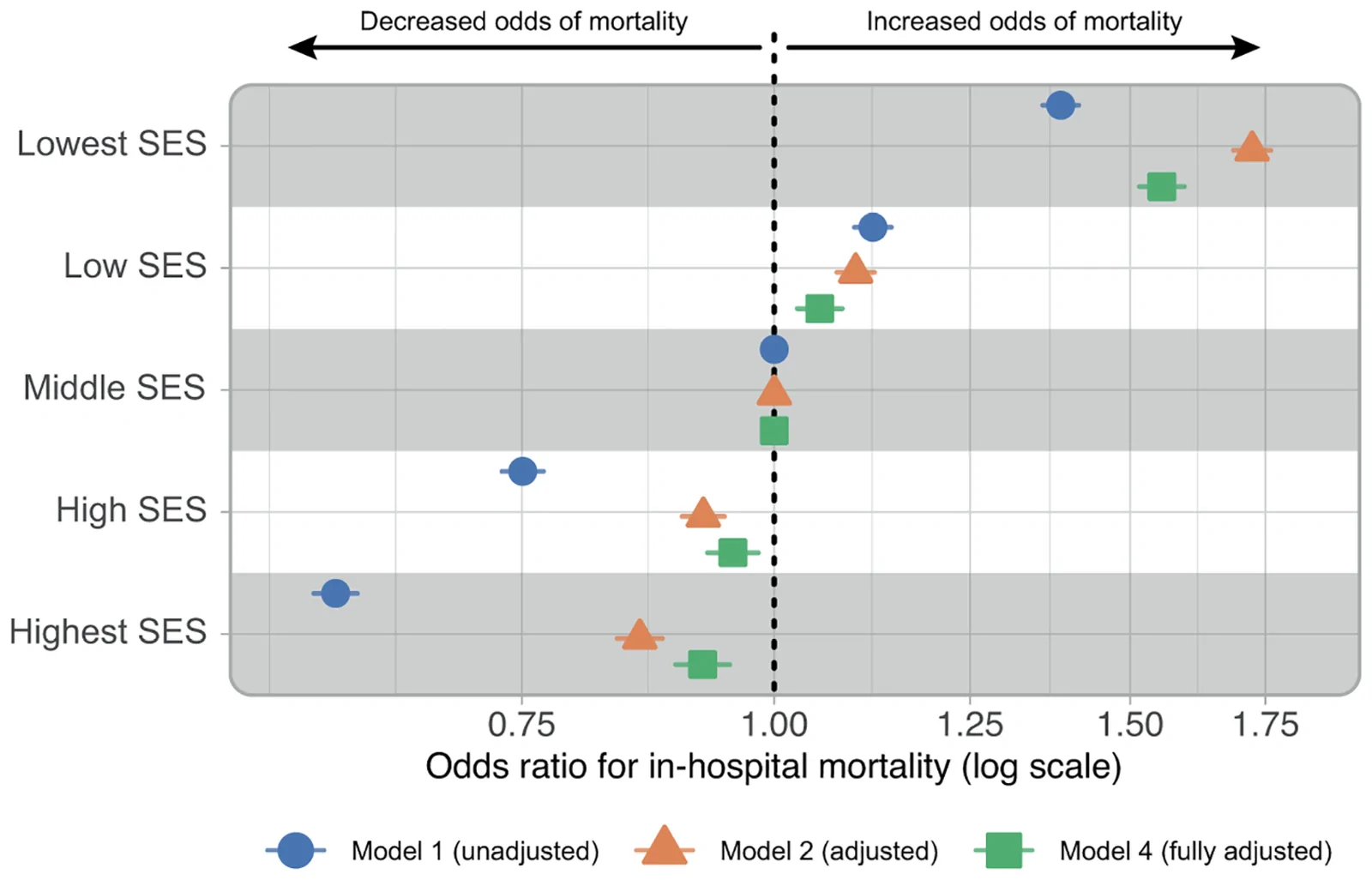
Odds ratios of hospital mortality for socioeconomic status (SES) quintiles with the middle SES as reference. Mixed-effects logistic regression models with ICU as a random intercept are displayed. Model 1 only included SES quintiles as determinants. Model 2 was adjusted for age, sex, and year of admission. Model 3 was additionally adjusted for individual comorbidities (Fig. 2). Model 4 was adjusted for age, sex, year of admission, all seven comorbidities, and the Acute Physiology Score. *Colors* and *shapes* indicate models; *horizontal bars* depict 95% CIs, and odds ratios are displayed on a logarithmic scale.

Results of models 3a–3g are presented in Tables S5–S11 (https://links.lww.com/CCM/I25). For all models with an individual comorbidity (models 3a–3g), a statistically significant interaction between SES and comorbidity was identified (*p* < 0.05), except for cardiovascular insufficiency (model 3d) and diabetes mellitus (model 3e), as determined by Wald and likelihood ratio tests (**Tables S14** and **S15**, https://links.lww.com/CCM/I25). The OR of hospital mortality for the lowest SES quintile decreased in the presence of respiratory insufficiency, renal insufficiency, cirrhosis, and immunological insufficiency (**Figs. 2** and **3**). Wald tests showed that the joint effect of the SES quintiles was associated with mortality in all models (Table S14, https://links.lww.com/CCM/I25), which remained statistically significant within each comorbidity stratum (**Table S16**, https://links.lww.com/CCM/I25). The sensitivity analysis showed that a higher number of comorbidities was associated with higher mortality and that the effect of SES quintiles was amplified in patients with two or more comorbidities (**Table S17**, https://links.lww.com/CCM/I25). The extreme-case sensitivity analyses showed slight differences in the ORs of the lowest and highest SES quintiles (**Tables S18** and **S19**, https://links.lww.com/CCM/I25).

**Figure 2.**
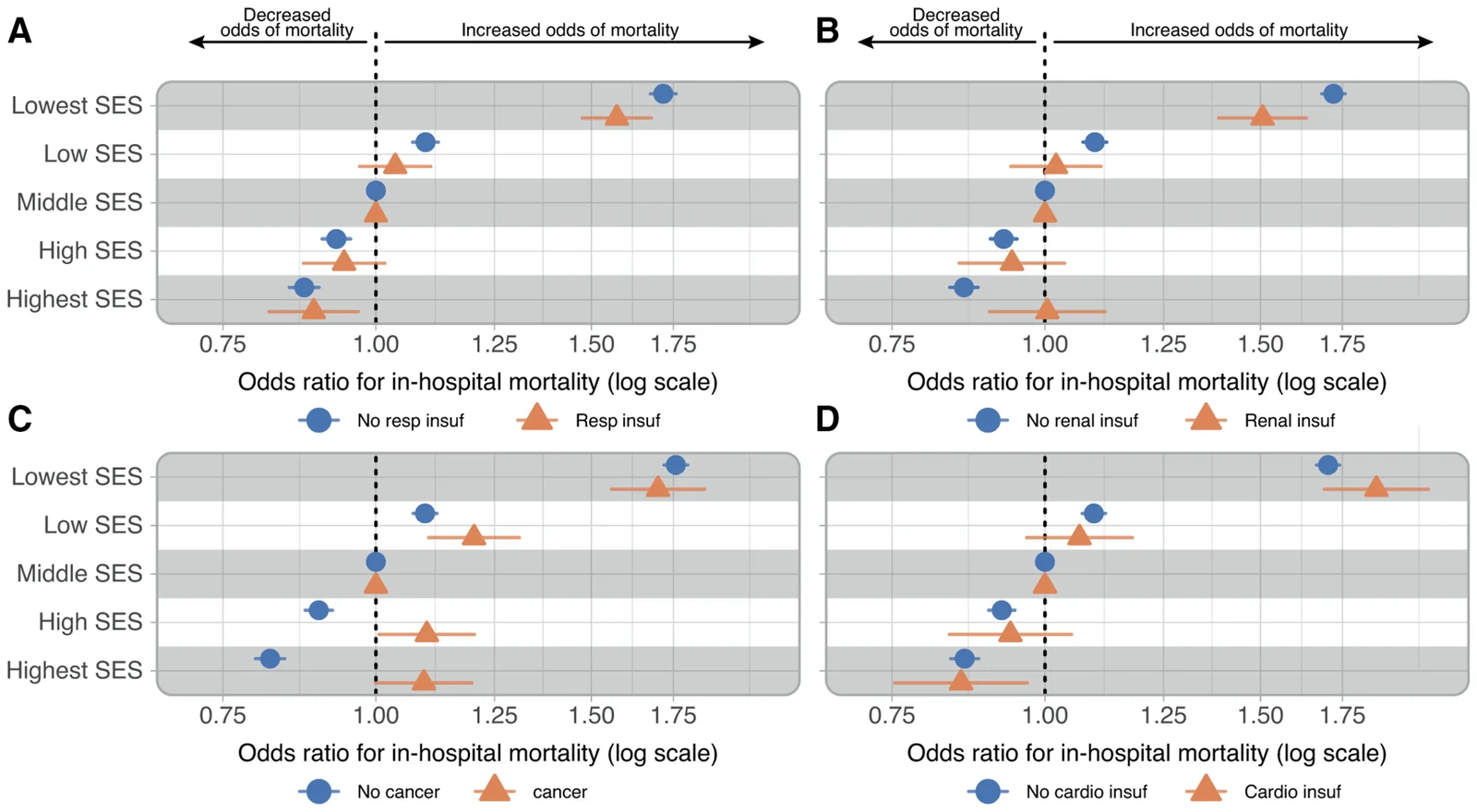
Odds ratios of hospital mortality for socioeconomic status (SES) quintiles with the middle SES as reference, stratified by comorbidity (models 3a–3d). Mixed-effects logistic regression models with ICU as a random intercept are displayed. Models were adjusted for age, sex, year, and comorbidity, and included an interaction term between SES and comorbidity. Stratified odds ratios were calculated from the interaction effect and are shown for presence (*orange triangles*) and absence (*blue dots*) of comorbidities (**A**–**D**) respiratory, renal, cancer, and cardiovascular. *Horizontal bars* indicate 95% CIs; odds ratios are displayed on a logarithmic scale. Resp = respiratory, Insuf = insufficiency, Cardio = cardiovascular.

**Figure 3.**
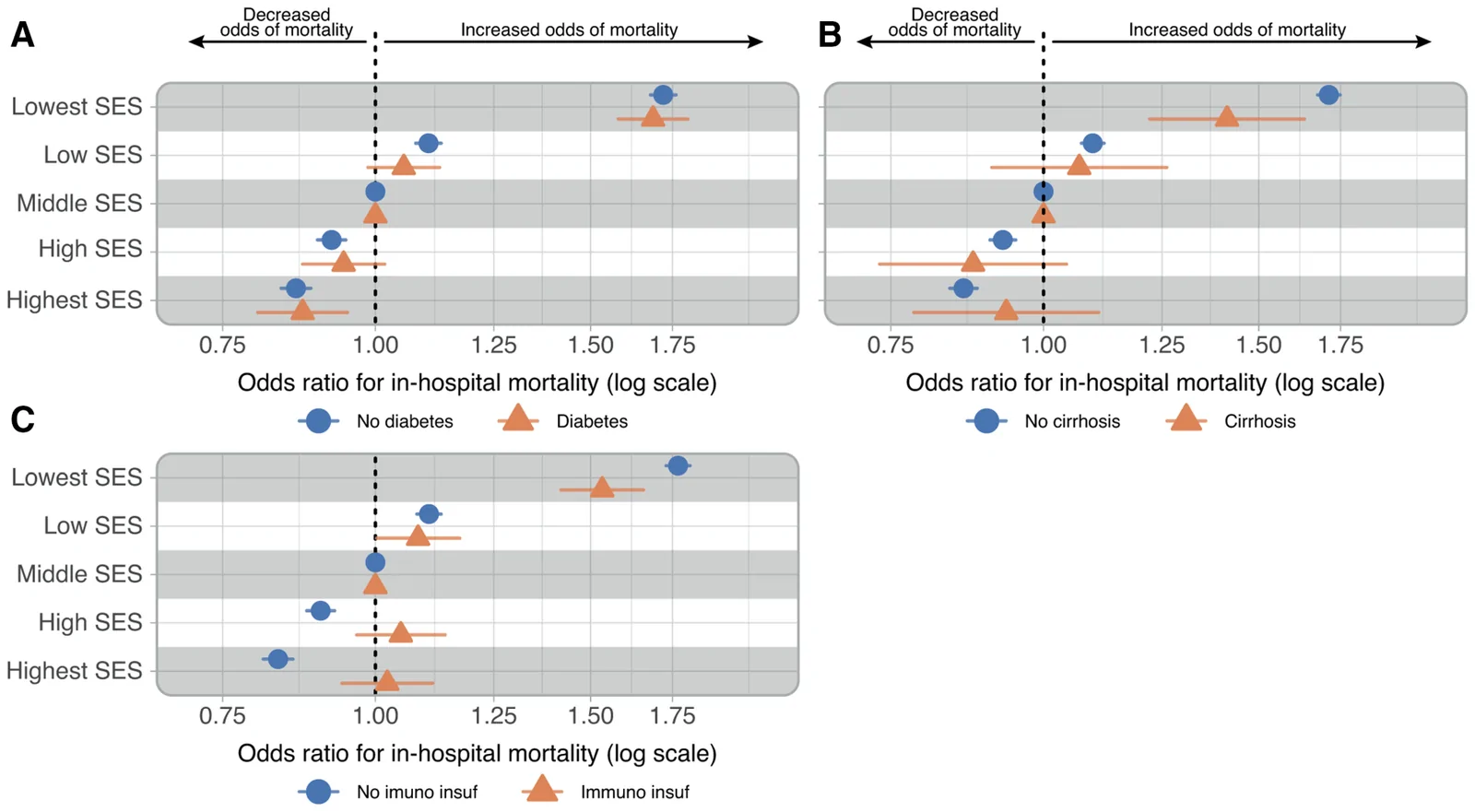
Odds ratios of hospital mortality for socioeconomic status (SES) quintiles with the middle SES as reference, stratified by comorbidity (models 3e–3g). Mixed-effects logistic regression models with ICU as a random intercept are displayed. Models were adjusted for age, sex, year, and comorbidity, and included an interaction term between SES and comorbidity. Stratified odds ratios were calculated from the interaction effect and are shown for presence (*orange triangles*) and absence (*blue dots*) of comorbidities (**A**–**C**) diabetes mellitus, cirrhosis, and immunological. *Horizontal bars* indicate 95% CIs; odds ratios are displayed on a logarithmic scale. Immuno = immunological, Insuf = insufficiency.

## DISCUSSION

In this nationwide cohort, we examined the relationship between SES and hospital mortality in patients admitted to Dutch ICUs. Lower SES was associated with higher hospital mortality and prevalence of comorbidities. The association between SES and hospital mortality persisted after adjusting for age, sex, year of ICU admission, APS, and measured comorbidities. This indicates that socioeconomic disparities cannot be fully explained by known comorbidities or acute disease severity. Compared to the middle SES quintile, the lowest quintile had the most pronounced higher odds of hospital mortality. Furthermore, each comorbidity modified the association between SES and mortality, except for cardiovascular insufficiency and diabetes mellitus. Stratification by individual comorbidities (models 3a–3g) showed that the strength of the association between SES and hospital mortality varied slightly across comorbidity groups. Nevertheless, the direction of the effect of the association remained consistent in the presence and absence of comorbidities. Taken together, this comprehensive study provides evidence that a lower SES is associated with higher mortality among Dutch ICU patients, despite the high quality, universally covered, and insurance-based Dutch healthcare system.

### Strengths and Limitations

The main strength of this study is the use of a large national cohort of ICU admissions, linked to high-quality household-level socioeconomic data. Routinely collected data from all Dutch ICUs provide nationally representative conclusions. The linkage of high-precision socioeconomic data with detailed clinical information offers a unique opportunity to examine the impact of SES on health outcomes within a universal healthcare system. This study also has some limitations. One potential limitation is collider-stratification bias, introduced by restricting the analyses to ICU patients. ICU admission is influenced by SES and other factors such as age, disease severity, and comorbidity. Conditioning at admission may therefore induce spurious associations between SES and these variables, potentially distorting the observed SES–mortality association. Adjustment by age, APS and comorbidities substantially altered the unadjusted associations, suggesting that much of the selection-related distortion was accounted for. However, residual bias from unmeasured causes of ICU admission and mortality cannot be fully ruled out. This primarily limits generalizability of our findings beyond the ICU setting. Moreover, the exclusion of hospitals that did not meet benchmark criteria could have resulted in selection bias. However, this exclusion is mostly related to temporary software or data transfer issues rather than structural differences in resources or patient populations. The cohort of excluded patients largely resembled the included patients’ SES distribution and hospital-type composition. Likewise, selection bias may have been introduced by excluding patients who could not be mapped or lacked SES information. However, this group was relatively small (5.0%) and represented socioeconomically advantaged and disadvantaged individuals (i.e., expatriates, tourists, [labor] migrants, and homeless individuals). Under the assumption that the ICU survival pattern of the excluded patients does not deviate strongly from the study population, and given the small proportion of exclusions, the impact on the results is likely negligible, as supported by the extreme-case sensitivity analyses. Complete case analysis (CCA) presents a minor limitation when data are missing at random. However, given our etiological focus and the lack of strong predictors for imputation, we preferred CCA combined with extreme-case sensitivity analysis to better understand the introduced bias. Although we adjusted for key demographic factors, comorbidities, and acute physiologic derangement, residual confounding remains a possibility, for instance, through lifestyle factors and unmeasured comorbidities. Moreover, the comorbidities were limited by their definitions (e.g., cardiovascular insufficiency was only classified as New York Heart Association Class IV (39)). Hence, patients with milder comorbidities were identified without any chronic conditions. The definition, measurement, distribution, and documentation of SES are country-specific; therefore, the generalizability of our results to other countries might be limited. Nevertheless, the direction of the present results is most likely universal, but with a different magnitude for each region or country. Furthermore, the present study does not include all social and demographic factors that may influence SES-related outcomes, such as immigration status, primary language, or ethnicity. Finally, although our analysis demonstrated clear associations between SES and mortality, the observational nature of our study prevents definitive causal conclusions from being drawn.

### Meaning of the Study

Several studies have reported higher mortality in lower SES groups (12–17), while others reported no statistically significant association (18–26). Differences in study design likely explain this heterogeneity, including how SES was conceptualized (e.g., income vs. wealth), the precision of SES measurement (e.g., individual vs. household vs. area-based SES), and sampling frames (e.g., restriction to specific hospitals). Although a recent systematic review concluded that, overall, lower SES is associated with higher mortality following ICU admission (11), the magnitude of this gradient may vary by healthcare system context and by how well SES captures lived social and material conditions. Evidence from nonuniversal systems (e.g., the United States) indicates that insurance coverage and healthcare access shape care pathways and outcomes (24, 27–29). However, in tax-funded universal systems (e.g., the United Kingdom, Canada, Australia, and Denmark), the SES gradient persists, despite universal coverage (13–17, 30). In (partly) insurance-based universal healthcare systems similar to the Netherlands (e.g., France and South Korea), most studies did not report a clear SES–mortality association (19–22, 31), possibly because those studies were typically small, single-center, or survey-based. Contrarily, the present nationwide analysis shows a clear gradient, providing evidence suggesting that universal insurance-based healthcare coverage is insufficient to eliminate socioeconomic health inequities.

Across prior studies reporting adjusted associations, confounder adjustment has altered the magnitude, and occasionally even the direction, of the SES–mortality association. However, the shape of the gradient typically remained stable, preserving the rank order across SES strata (14, 15, 24, 30, 33). In contrast, we show SES-group-specific changes after confounder adjustments, which revealed excess risk concentrated in the lowest SES quintile. Moreover, prior ICU studies typically treated comorbidity as a confounder, often via composite indices (14, 15, 17, 18, 22–25, 30, 31, 33, 37), and reported a higher prevalence of comorbidities in lower SES groups (18, 46–52). However, comorbidity-specific effect modification of the SES–mortality association appeared understudied. The present study adds evidence by using comorbidity-specific interaction terms that revealed detailed modifying effects across comorbidities. Nevertheless, the overall SES–mortality gradient persisted in both the presence and absence of comorbidities.

In the present investigation, the differences in baseline characteristics between SES quintiles were striking. The lowest SES quintile was younger on average than the low and middle SES quintiles. This is consistent with other studies and could be explained by premature aging, lower lifespan, and collider-stratification bias (53–56). Lower SES quintiles also consisted of more women and severely ill patients, as indicated by the higher APS. Similar to previous studies, we observed a higher prevalence of respiratory insufficiency, renal insufficiency, diabetes mellitus, and cirrhosis in the lower SES quintiles (14, 52, 57–60). In contrast, cancer was less prevalent in lower SES quintiles. Previous studies show that the prevalence of prostate, breast, and colon cancer is lower in low socioeconomic groups (61, 62), whereas colorectal, lung, stomach, oropharyngeal, and esophageal cancers are more prevalent in low socioeconomic groups (62–64). This highlights the complex nature of the association between SES and comorbidity, with many potentially contributing factors, such as lifestyle, healthcare avoidance, illiteracy, and culture.

The association between SES and hospital mortality persisted after adjusting for baseline characteristics, though the causal pathways remain complex. Even within universal healthcare systems, lower SES individuals face cumulative access barriers, illustrated for example by lower preventive screening participation (65, 66). Navigating complex care pathways repeatedly discourages engagement, possibly delaying care until ICU admission becomes necessary (67–69). Importantly, these mechanisms vary across healthcare systems. In the Dutch general practitioner gatekeeping model, capitation-based funding can restrict capacity in socioeconomically deprived areas. Also, lower health literacy impedes effective patient-provider communication. Furthermore, anticipated out-of-pocket costs from mandatory annual deductibles may discourage or delay specialist referrals (70). Furthermore, lacking financial resources obstructs preventative care, treatment adherence, and healthy lifestyles. At the hospital level, socioeconomic factors may further complicate communication between patients and care providers or social support (71, 72). Although challenging to quantify, these factors likely contribute to the health disparities observed in our study.

We observed a smaller effect size in patients with respiratory insufficiency, renal insufficiency, cancer, diabetes mellitus, and immunological insufficiency than in those without these conditions. This may reflect that patients with chronic comorbidities are often more engaged with the healthcare system. Nevertheless, the stratified analyses showed that the socioeconomic differences persisted in patients with and without specific comorbidities.

We revealed a substantial health disparity and suggest that policymakers should aid healthcare professionals in mitigating its detrimental effects. Healthcare professionals are uniquely positioned to identify patients from lower socioeconomic backgrounds. Although they cannot directly address financial or societal risk factors, they can improve outcomes through tailored communication that overcomes language and knowledge barriers (71, 73, 74). Given the sensitive nature of financial discussions, time constraints, and limited training, healthcare professionals should be supported with targeted education, communication protocols, and treatment plans (72, 75). Furthermore, socioeconomic factors should be considered when comparing ICU performance so ICUs caring for more socioeconomically disadvantaged patients are not unfairly penalized in performance assessments.

### Future Research

Further research is needed to disentangle which components of SES (i.e., wealth, education, or employment) drive disparities in ICU outcomes. This may help identify mechanisms of vulnerability and provide opportunities for targeted interventions. In addition, we urge researchers to perform similar analyses to assess whether the associations between SES, comorbidities, and hospital mortality are consistent across countries and healthcare systems. Future work should also explore how pre-ICU care, communication barriers, and ICU admission practices may impact these associations.

## CONCLUSIONS

In this nationwide study using precise household-level socioeconomic data, lower SES was independently associated with higher mortality among ICU patients after adjusting for demographic factors, comorbidities, and acute physiologic derangement. These findings highlight the impact of social determinants of health on critical care outcomes, underscoring the need for effective and equitable care. Regional differences in socioeconomic composition and the impact of SES on outcomes should inform policymakers and healthcare professionals regarding prevention strategies and care delivery. Addressing these disparities is essential for improving outcomes in critical illness and ensuring consistent access to high-quality care.

## ACKNOWLEDGMENTS

We thank all the ICUs for their efforts in providing high-quality data to the National Intensive Care Evaluation (NICE) registry and for making these data available for our study. We would also like to thank in particular Statistics Netherlands and NICE Research and Support for their assistance in the linkage process. This project was funded by the Board of Directors of Maastricht University Medical Centre+ and the National Intensive Care Evaluation.

## Supplementary Material
