# Supplementary material for: Socioeconomic Disparities and the Role of Comorbidity in Hospital Mortality: A Dutch Nationwide Critical Care Cohort Study
Source: Crit Care Med. 2026 Jul 21;54(10):2754–65. doi: 10.1097/CCM.0000000000007271 (PMC13632809; doi:10.1097/CCM.0000000000007271)
Supplement: Supplementary file 1 [file ccm-54-2754-s001.pdf]

Supplementary Materials - Socioeconomic disparities and the role of comorbidity in hospital mortality: A Dutch nationwide critical care cohort study

Table of contents

Abbreviations 1

Methods 2

Results 2

Table S1: Overview of the models fitted in this study. 2

Table S2: Descriptive statistics of the original data, each individual exclusion step, and the study population. 3

Table S3: Model 1 results 4

Table S4: Model 2 results 4

Table S5: Model 3a results 5

Table S6: Model 3b results 5

Table S7: Model 3c results 6

Table S8: Model 3d results 6

Table S9: Model 3e results. 7

Table S10: Model 3f results 7

Table S11: Model 3g results 8

Table S12: Model 4 results 8

Table S13: Model comparison based on fit statistics. 9

Table S14: Wald test results of all models. 9

Table S15: Likelihood Ratio Test (LRT) results of the interaction terms of the interaction models (Model 3 a-g). 11

Table S16: Wald test results for the joint effect of SES stratified by comorbidity, based on the interaction models (Model 3 a-g). 11

Table S17: Sensitivity analysis of model 3 with the number of comorbidities instead of a specific comorbidity 12

Table S18: Extreme-case sensitivity analysis of model 4 where the minimum SES was imputed for patients that were not linked or were missing SES 12

Table S19: Extreme-case sensitivity analysis of model 4 where the maximum SES was imputed for patients that were not linked or were missing SES 13

References 13

Abbreviations

SES: socioeconomic status

CI: Confidence interval

AIC: Akaike information criterion

BIC: Bayesian information criterion

df: degrees of freedom

Methods

The SES score is based on component scores for wealth, education level, and employment history. The employment history is based on the main financial provider over the past four years, while the educational level describes the highest level of education achieved by the main financial provider or a registered partner. The wealth component corresponds to the household's total assets (e.g. savings, real estate, and debts) and standardised spendable income adjusted for household size and composition using the Organisation for Economic Co-operation and Development (OECD) equivalence scale. To minimise the impact of missing SES data, we used SES scores from the year preceding ICU admission, or if unavailable, from two years preceding ICU admission. Admissions that met the APACHE IV exclusion criteria (41) were excluded. For instance, only the first admission of patients who were readmitted to the ICU without discharge from the hospital was included. Data that did not fulfil the benchmark criteria were excluded. Benchmark criteria are checked during NICE site visits by comparing a random sample of electronic health records (EHRs) against the submitted data. If an ICU did not meet the NICE benchmark criteria, due to excessive missing data or data not conforming to NICE definitions, its data were excluded from the research datasets for at least one year until the data quality was proven to be sufficient. The SES score was categorised into quintiles, with corresponding ranges from lowest to highest of −2.330 to −0.831, −0.828 to −0.276, −0.271 to 0.155, 0.163 to 0.484, and 0.506 to 1.036, respectively.

The following seven comorbidities were available in the dataset: (1) respiratory insufficiency, defined as patients with medication for chronic obstructive pulmonary disease (COPD) or oxygen dependence at home (excluding sleep apnoea); (2) renal insufficiency, defined as patients with long-term haemodialysis, peritoneal dialysis, or renal insufficiency with creatinine levels exceeding 177 μmol/L; (3) cancer, defined as patients with malignant lymphoma, acute leukaemia, multiple myeloma, diagnosed metastases, or stage IV cancer; (4) cardiovascular insufficiency, defined as patients with New York Heart Association (NYHA) class IV heart failure or angina with symptoms at rest; (5) diabetes mellitus, defined as patients with type 1 or type 2 diabetes mellitus requiring medication at home; (6) cirrhosis, defined as patients with a positive liver biopsy or scan and documented portal hypertension, or portal hypertension resulting in periods of high gastrointestinal bleeding, or previous periods of hepatic failure, coma, or encephalopathy; and (7) immunological insufficiency, defined as patients with acquired immune deficiency syndrome (AIDS), documented humoral or cellular deficiencies, or those receiving long-term immunosuppressive therapy including specific corticosteroid dosages, hydroxyurea treatment, or chemotherapy, or radiotherapy within the past year specifically for lymphoma management requiring ICU admission (1–3).

Results

Table S1: Overview of the models fitted in this study.

Model	Independent variable(s)
**Model 1**	SES[1]
**Model 2**	SES[1] + age + sex + year[2]
**Model 3 (a-g)**[3]	SES[1] + age + sex + year[2] + comorbidity(a-g) + comorbidity(a-g) * SES[1]
**Model 4**	SES[1] + age + sex + year[2] + APS + all comorbidities

SES=socioeconomic status. APS=Acute Physiology Score. ICU=intensive care unit.

1. SES quintiles as a categorical variable
2. Year of ICU admission
3. These models were fitted individually for each comorbidity a to g

Table S2: Descriptive statistics of the original data, each individual exclusion step, and the study population.

Variable	All NICE admissions	APACHE IV exclusion	Benchmark exclusion	Not mapped	No SES data available	Study population
**Admissions**, N	703,773	64,792	15,143	18,082	17,188	588,568
**SES**, mean (SD)	-0.19 (0.77)	-0.22 (0.77)	-0.15 (0.76)	NA	NA	-0.19 (0.77)
Wealth component	-0.03 (0.37)	-0.05 (0.37)	-0.02 (0.36)	NA	NA	-0.03 (0.36)
Education component	-0.08 (0.31)	-0.09 (0.31)	-0.05 (0.30)	NA	NA	-0.08 (0.31)
Employment comp.	-0.08 (0.32)	-0.09 (0.32)	-0.08 (0.32)	NA	NA	-0.08 (0.32)
**Age**, mean (SD)	63.1 (15.7)	63.2 (15.8)	63.5 (15.6)	54.0 (17.8)	58.8 (20.1)	63.5 (15.3)
**Sex** (male), N (%)	857,238 (60.9%)	39,204 (60.5%)	9,011 (59.5%)	11,555 (64.0%)	10,002 (58.2%)	358,847 (61.0%)
N-Miss	82	6	0	35	0	0
**BMI**, mean (SD)	26.9 (5.5)	26.5 (5.6)	27.1 (5.8)	26.6 (5.8)	26.4 (6.2)	26.9 (5.5)
**Admission type**, N (%)						
N-Miss	4,984	2,492	0	0	0	0
Deceased	1,360 (0.1%)	680 (1.1%)	0 (0.0%)	0 (0.0%)	0 (0.0%)	0 (0.0%)
Medical	746,700 (53.2%)	41,790 (67.1%)	8,411 (55.5%)	11,409 (63.1%)	12,365 (71.9%)	299,375 (50.9%)
Emergency surgical	166,704 (11.9%)	11,045 (17.7%)	1,412 (9.3%)	2,577 (14.3%)	2,077 (12.1%)	66,241 (11.3%)
Planned surgical	487,798 (34.8%)	8,785 (14.1%)	5,320 (35.1%)	4,096 (22.7%)	2,746 (16.0%)	222,952 (37.9%)
**Planned admission**, N (%)	438,240 (31.1%)	7,807 (12.0%)	4,855 (32.1%)	3,730 (20.6%)	2,301 (13.4%)	200,427 (34.1%)
**Volume group**, N (%)						
Very small	63,292 (4.5%)	3,109 (4.8%)	1,632 (10.8%)	1,012 (5.6%)	883 (5.1%)	25,010 (4.2%)
Small	124,892 (8.9%)	6,174 (9.5%)	1,725 (11.4%)	1,695 (9.4%)	1,905 (11.1%)	50,947 (8.7%)
Medium	198,516 (14.1%)	8,194 (12.6%)	2,34 (1.5%)	2,428 (13.4%)	2,986 (17.4%)	85,416 (14.5%)
Large	328,348 (23.3%)	14,358 (22.2%)	4,308 (28.4%)	4,209 (23.3%)	4,662 (27.1%)	136,637 (23.2%)
Very large	692,498 (49.2%)	32,957 (50.9%)	7,244 (47.8%)	8,738 (48.3%)	6,752 (39.3%)	290,558 (49.4%)
**Hospital type**, N (%)						
General peripheral hospital	400,392 (28.4%)	18,951 (29.2%)	3,591 (23.7%)	5,741 (31.7%)	5,844 (34.0%)	166,069 (28.2%)
Teaching hospital (non-academic)	686,656 (48.8%)	27,947 (43.1%)	7,366 (48.6%)	7,543 (41.7%)	8,154 (47.4%)	292,318 (49.7%)
Teaching hospital (academic)	320,498 (22.8%)	17,894 (27.6%)	4,186 (27.6%)	4,798 (26.5%)	3,190 (18.6%)	130,181 (22.1%)
**APS score**, mean (SD)	45.0 (26.3)	47.4 (30.2)	38.8 (25.2)	47.1 (28.0)	49.6 (27.3)	44.7 (25.8)
**APACHE IV mortality risk**, *mean (SD)*	17.2% (22.8)	26.4% (26.4)	13.8% (19.8)	17.3% (23.0)	19.0% (22.6)	16.3% (22.2)
**Chronic diagnoses**, N (%)						
None	812,036 (57.7%)	36,296 (56.0%)	8,947 (59.1%)	11,632 (64.3%)	10,013 (58.3%)	339,130 (57.6%)
One	406,996 (28.9%)	18,010 (27.8%)	4,312 (28.5%)	4,372 (24.2%)	5,100 (29.7%)	171,704 (29.2%)
More than one	188,514 (13.4%)	10,486 (16.2%)	1,884 (12.4%)	2,078 (11.5%)	2,075 (12.1%)	77,734 (13.2%)
**ICU LOS** (24 hours), mean (SD)	3.4 (7.4)	3.7 (9.8)	3.4 (7.6)	4.4 (9.1)	3.3 (6.3)	3.3 (7.0)
**Hospital LOS** (calendar days), mean (SD)	13.3 (17.0)	24.6 (29.9)	12.6 (16.0)	14.0 (20.2)	10.6 (13.8)	12.1 (14.5)
**Hospital mortality**, N (%)	186,076 (13.2%)	14,452 (22.3%)	2,004 (13.2%)	2,029 (11.2%)	3,074 (17.9%)	71,479 (12.1%)

Table S3: Model 1 results. SES quintile 3 was used as reference category.

Variable	Odds Ratio (95% CI)	z-Statistic	p-value
(Intercept)	0.14 (0.13-0.15)	-50.89	0.00
SES quintile 1	1.39 (1.35-1.42)	27.68	0.00
SES quintile 2	1.12 (1.09-1.15)	9.30	0.00
SES quintile 4	0.75 (0.73-0.77)	-21.38	0.00
SES quintile 5	0.61 (0.59-0.62	-35.75	0.00

Table S4: Model 2 results. SES quintile 3 and admission year 2019 were used as reference categories.

Variable	Odds Ratio (95% CI)	z-Statistic	p-value
(Intercept)	0.10 (0.09-0.11)	-52.28	0.00
SES quintile 1	1.72 (1.68-1.77)	44.69	0.00
SES quintile 2	1.10 (1.07-1.12)	7.54	0.00
SES quintile 4	0.92 (0.90-0.95)	-5.89	0.00
SES quintile 5	0.86 (0.83-0.88)	-10.55	0.00
Age (standardised)	1.73 (1.72-1.75)	106.23	0.00
Sex (male)	1.01 (0.99-1.02)	0.86	0.39
Year of admission (2014)	0.96 (0.93-0.99)	-2.43	0.02
Year of admission (2015)	1.01 (0.98-1.05)	0.78	0.43
Year of admission (2016)	1.02 (0.98-1.05)	1.07	0.29
Year of admission (2017)	1.00 (0.97-1.04)	0.27	0.79
Year of admission (2018)	1.05 (1.02-1.09)	2.86	0.00
Year of admission (2020)	1.18 (1.14-1.22)	9.14	0.00
Year of admission (2021)	1.28 (1.24-1.33)	14.01	0.00
Year of admission (2022)	1.13 (1.09-1.17)	6.55	0.00

Table S5: Model 3a results. SES quintile 3 and admission year 2019 were used as reference categories.

Variable	Odds Ratio (95% CI)	z-Statistic	p-value
(Intercept)	0.09 (0.08–0.10)	-52.56	0.00
SES quintile 1	1.72 (1.67–1.76)	39.38	0.00
SES quintile 2	1.10 (1.07–1.13)	7.23	0.00
SES quintile 4	0.93 (0.90–0.96)	-5.44	0.00
SES quintile 5	0.87 (0.85–0.90)	-9.48	0.00
Age (standardised)	1.72 (1.70–1.74)	105.17	0.00
Sex (male)	1.01 (0.99–1.03)	0.58	0.56
Year of admission (2014)	0.96 (0.93–1.00)	-2.39	0.02
Year of admission (2015)	1.02 (0.98–1.05)	0.82	0.41
Year of admission (2016)	1.02 (0.99–1.06)	1.09	0.28
Year of admission (2017)	1.01 (0.97–1.04)	0.32	0.75
Year of admission (2018)	1.05 (1.02–1.09)	2.86	0.00
Year of admission (2020)	1.19 (1.15–1.24)	9.12	0.00
Year of admission (2021)	1.31 (1.26–1.35)	13.99	0.00
Year of admission (2022)	1.14 (1.10–1.18)	6.55	0.00
Respiratory insufficiency (present)	1.53 (1.47–1.60)	4.85	0.00
SES quintile 1 × respiratory insufficiency	0.92 (0.87–0.97)	-0.67	0.51
SES quintile 2 × respiratory insufficiency	0.94 (0.89–1.00)	-1.55	0.12
SES quintile 4 × respiratory insufficiency	1.01 (0.95–1.09)	0.64	0.52
SES quintile 5 × respiratory insufficiency	1.02 (0.94–1.10)	0.32	0.75

Table S6: Model 3b results. SES quintile 3 and admission year 2019 were used as reference categories.

Variable	Odds Ratio (95% CI)	z-Statistic	p-value
(Intercept)	0.10 (0.09–0.10)	-53.73	0.00
SES quintile 1	1.72 (1.68–1.76)	42.24	0.00
SES quintile 2	1.10 (1.07–1.13)	7.23	0.00
SES quintile 4	0.93 (0.90–0.95)	-5.38	0.00
SES quintile 5	0.86 (0.83–0.88)	-10.07	0.00
Age (standardised)	1.71 (1.69–1.72)	102.66	0.00
Sex (male)	0.99 (0.98–1.01)	-0.76	0.45
Year of admission (2014)	0.96 (0.93–1.00)	-2.03	0.04
Year of admission (2015)	1.02 (0.99–1.06)	1.17	0.24
Year of admission (2016)	1.02 (0.99–1.06)	1.34	0.18
Year of admission (2017)	1.01 (0.98–1.04)	0.52	0.61
Year of admission (2018)	1.06 (1.02–1.09)	3.07	0.00
Year of admission (2020)	1.19 (1.15–1.23)	9.65	0.00
Year of admission (2021)	1.30 (1.26–1.35)	14.80	0.00
Year of admission (2022)	1.14 (1.10–1.18)	7.23	0.00
Renal insufficiency (present)	1.88 (1.77–1.99)	20.82	0.00
SES quintile 1 × renal insufficiency	0.88 (0.81–0.95)	-3.36	0.00
SES quintile 2 × renal insufficiency	0.93 (0.86–1.01)	-1.79	0.07
SES quintile 4 × renal insufficiency	1.02 (0.92–1.12)	0.32	0.75
SES quintile 5 × renal insufficiency	1.17 (1.05–1.30)	2.93	0.00

Table S7: Model 3c results. SES quintile 3 and admission year 2019 were used as reference categories.

Variable	Odds Ratio (95% CI)	z-Statistic	p-value
(Intercept)	0.09 (0.08–0.10)	-52.37	0.00
SES quintile 1	1.76 (1.71–1.80)	44.09	0.00
SES quintile 2	1.10 (1.07–1.13)	7.10	0.00
SES quintile 4	0.90 (0.87–0.92)	-7.34	0.00
SES quintile 5	0.82 (0.79–0.84)	-12.79	0.00
Age (standardised)	1.73 (1.71–1.75)	104.51	0.00
Sex (male)	1.01 (0.99–1.03)	1.22	0.22
Year of admission (2014)	0.96 (0.93–1.00)	-2.05	0.04
Year of admission (2015)	1.02 (0.99–1.06)	1.12	0.26
Year of admission (2016)	1.02 (0.99–1.06)	1.39	0.17
Year of admission (2017)	1.01 (0.98–1.05)	0.63	0.53
Year of admission (2018)	1.06 (1.02–1.09)	3.08	0.00
Year of admission (2020)	1.19 (1.15–1.23)	9.63	0.00
Year of admission (2021)	1.31 (1.26–1.36)	15.06	0.00
Year of admission (2022)	1.14 (1.10–1.18)	7.24	0.00
Cancer (present)	2.30 (2.17–2.44)	28.54	0.00
SES quintile 1 × cancer	0.97 (0.89–1.05)	-0.79	0.43
SES quintile 2 × cancer	1.10 (1.01–1.19)	2.25	0.02
SES quintile 4 × cancer	1.23 (1.13–1.33)	4.73	0.00
SES quintile 5 × cancer	1.34 (1.23–1.45)	6.70	0.00

Table S8: Model 3d results. SES quintile 3 and admission year 2019 were used as reference categories.

Variable	Odds Ratio (95% CI)	z-Statistic	p-value
(Intercept)	0.10 (0.09–0.11)	-52.13	0.00
SES quintile 1	1.70 (1.66–1.75)	42.27	0.00
SES quintile 2	1.10 (1.07–1.12)	7.22	0.00
SES quintile 4	0.92 (0.90–0.95)	-5.73	0.00
SES quintile 5	0.86 (0.84–0.89)	-10.12	0.00
Age (standardised)	1.72 (1.70–1.74)	104.24	0.00
Sex (male)	1.00 (0.98–1.02)	0.07	0.94
Year of admission (2014)	0.95 (0.92–0.99)	-2.78	0.01
Year of admission (2015)	1.01 (0.98–1.05)	0.72	0.47
Year of admission (2016)	1.02 (0.98–1.05)	1.05	0.29
Year of admission (2017)	1.01 (0.97–1.04)	0.43	0.67
Year of admission (2018)	1.05 (1.02–1.09)	2.96	0.00
Year of admission (2020)	1.18 (1.14–1.22)	9.41	0.00
Year of admission (2021)	1.29 (1.25–1.34)	14.39	0.00
Year of admission (2022)	1.13 (1.09–1.17)	6.86	0.00
Cardiovascular insufficiency (present)	1.57 (1.46–1.69)	12.49	0.00
SES quintile 1 × cardiovascular insufficiency	1.10 (1.00–1.20)	1.89	0.06
SES quintile 2 × cardiovascular insufficiency	0.97 (0.88–1.07)	-0.55	0.58
SES quintile 4 × cardiovascular insufficiency	1.02 (0.91–1.14)	0.28	0.78
SES quintile 5 × cardiovascular insufficiency	0.99 (0.88–1.12)	-0.11	0.91

Table S9: Model 3e results. SES quintile 3 and admission year 2019 were used as reference categories.

Variable	Odds Ratio (95% CI)	z-Statistic	p-value
(Intercept)	0.10 (0.09–0.11)	-52.56	0.00
SES quintile 1	1.72 (1.67–1.77)	39.38	0.00
SES quintile 2	1.11 (1.08–1.14)	7.23	0.00
SES quintile 4	0.92 (0.89–0.95)	-5.44	0.00
SES quintile 5	0.86 (0.83–0.89)	-9.48	0.00
Age (standardised)	1.73 (1.71–1.75)	105.17	0.00
Sex (male)	1.00 (0.99–1.02)	0.58	0.56
Year of admission (2014)	0.96 (0.93–0.99)	-2.39	0.02
Year of admission (2015)	1.01 (0.98–1.05)	0.82	0.41
Year of admission (2016)	1.02 (0.98–1.05)	1.09	0.28
Year of admission (2017)	1.01 (0.97–1.04)	0.32	0.75
Year of admission (2018)	1.05 (1.02–1.09)	2.86	0.00
Year of admission (2020)	1.18 (1.14–1.22)	9.12	0.00
Year of admission (2021)	1.28 (1.24–1.33)	13.99	0.00
Year of admission (2022)	1.13 (1.09–1.17)	6.55	0.00
Diabetes mellitus (present)	1.11 (1.07–1.16)	4.85	0.00
SES quintile 1 × diabetes mellitus	0.98 (0.93–1.04)	-0.67	0.51
SES quintile 2 × diabetes mellitus	0.95 (0.90–1.01)	-1.55	0.12
SES quintile 4 × diabetes mellitus	1.02 (0.95–1.10)	0.64	0.52
SES quintile 5 × diabetes mellitus	1.01 (0.94–1.09)	0.32	0.75

Table S10: Model 3f results. SES quintile 3 and admission year 2019 were used as reference categories.

Variable	Odds Ratio (95% CI)	z-Statistic	p-value
(Intercept)	0.10 (0.09–0.10)	-53.39	0.00
SES quintile 1	1.71 (1.67–1.75)	43.44	0.00
SES quintile 2	1.10 (1.07–1.12)	7.44	0.00
SES quintile 4	0.93 (0.90–0.95)	-5.51	0.00
SES quintile 5	0.86 (0.84–0.89)	-10.23	0.00
Age (standardised)	1.76 (1.74–1.78)	107.78	0.00
Sex (male)	1.00 (0.98–1.02)	0.07	0.94
Year of admission (2014)	0.96 (0.93–1.00)	-2.18	0.03
Year of admission (2015)	1.02 (0.98–1.05)	1.08	0.28
Year of admission (2016)	1.02 (0.99–1.06)	1.36	0.17
Year of admission (2017)	1.01 (0.98–1.05)	0.57	0.57
Year of admission (2018)	1.05 (1.02–1.09)	2.98	0.00
Year of admission (2020)	1.18 (1.14–1.22)	9.40	0.00
Year of admission (2021)	1.30 (1.25–1.34)	14.58	0.00
Year of admission (2022)	1.13 (1.09–1.17)	6.68	0.00
Cirrhosis (present)	3.89 (3.45–4.37)	22.47	0.00
SES quintile 1 × cirrhosis	0.83 (0.71–0.95)	-2.60	0.01
SES quintile 2 × cirrhosis	0.97 (0.83–1.15)	-0.30	0.76
SES quintile 4 × cirrhosis	0.95 (0.79–1.13)	-0.63	0.53
SES quintile 5 × cirrhosis	1.08 (0.91–1.29)	0.91	0.36

Table S11: Model 3g results. SES quintile 3 and admission year 2019 were used as reference categories.

Variable	Odds Ratio (95% CI)	z-Statistic	p-value
(Intercept)	0.09 (0.08–0.10)	-53.85	0.00
SES quintile 1	1.77 (1.72–1.81)	43.75	0.00
SES quintile 2	1.11 (1.08–1.14)	7.64	0.00
SES quintile 4	0.90 (0.88–0.93)	-6.93	0.00
SES quintile 5	0.83 (0.81–0.86)	-11.64	0.00
Age (standardised)	1.74 (1.73–1.76)	105.89	0.00
Sex (male)	1.02 (1.00–1.03)	1.89	0.06
Year of admission (2014)	0.98 (0.94–1.01)	-1.40	0.16
Year of admission (2015)	1.03 (1.00–1.07)	1.71	0.09
Year of admission (2016)	1.03 (1.00–1.07)	1.91	0.06
Year of admission (2017)	1.02 (0.99–1.06)	1.24	0.22
Year of admission (2018)	1.06 (1.03–1.10)	3.40	0.00
Year of admission (2020)	1.19 (1.15–1.23)	9.61	0.00
Year of admission (2021)	1.30 (1.25–1.35)	14.64	0.00
Year of admission (2022)	1.14 (1.10–1.18)	7.06	0.00
Immunological insufficiency (present)	2.13 (2.02–2.24)	28.58	0.00
SES quintile 1 × immunological insufficiency	0.87 (0.81–0.93)	-3.90	0.00
SES quintile 2 × immunological insufficiency	0.98 (0.91–1.05)	-0.56	0.58
SES quintile 4 × immunological insufficiency	1.16 (1.08–1.26)	3.86	0.00
SES quintile 5 × immunological insufficiency	1.23 (1.14–1.33)	5.13	0.00

Table S12: Model 4 results. SES quintile 3 and admission year 2019 were used as reference categories.

Variable	Odds Ratio (95% CI)	z-Statistic	p-value
(Intercept)	0.04 (0.04–0.05)	-79.00	0.00
SES quintile 1	1.56 (1.51–1.60)	30.55	0.00
SES quintile 2	1.05 (1.02–1.08)	3.58	0.00
SES quintile 4	0.95 (0.92–0.98)	-2.92	0.00
SES quintile 5	0.92 (0.89–0.95)	-4.79	0.00
Age (standardised)	1.80 (1.78–1.82)	92.63	0.00
Sex (male)	0.99 (0.97–1.01)	-1.19	0.23
Year of admission (2014)	1.02 (0.98–1.06)	1.06	0.29
Year of admission (2015)	1.07 (1.03–1.11)	3.27	0.00
Year of admission (2016)	1.08 (1.04–1.13)	3.83	0.00
Year of admission (2017)	1.01 (0.97–1.05)	0.44	0.66
Year of admission (2018)	1.05 (1.00–1.09)	2.19	0.03
Year of admission (2020)	1.26 (1.21–1.32)	11.24	0.00
Year of admission (2021)	1.45 (1.39–1.51)	17.71	0.00
Year of admission (2022)	1.18 (1.13–1.23)	7.76	0.00
Acute Physiology Score (APS) (standardised)	3.72 (3.68–3.75)	270.49	0.00
Respiratory insufficiency (present)	1.63 (1.59–1.67)	40.43	0.00
Renal insufficiency (present)	1.00 (0.97–1.04)	0.22	0.83
Cancer (present)	2.12 (2.04–2.19)	43.09	0.00
Cardiovascular insufficiency (present)	1.48 (1.42–1.54)	19.12	0.00
Diabetes mellitus (present)	0.89 (0.87–0.91)	-9.76	0.00
Cirrhosis (present)	2.13 (2.00–2.26)	24.50	0.00
Immunological insufficiency (present)	1.50 (1.45–1.54)	25.99	0.00

Table S13: Model comparison based on fit statistics.

Model	AIC	BIC	Log-likelihood	Residual df
model 1	423982.43	424050.14	-211985.22	588562.00
model 2	410483.44	410664.01	-205225.72	588552.00
model 3a	409084.13	409321.12	-204521.06	588547.00
model 3b	408872.97	409109.97	-204415.49	588547.00
model 3c	406366.62	406603.62	-203162.31	588547.00
model 3d	409776.15	410013.15	-204867.08	588547.00
model 3e	410402.91	410639.91	-205180.46	588547.00
model 3f	408326.01	408563.00	-204142.00	588547.00
model 3g	406845.49	407082.49	-203401.75	588547.00
model 4	297442.49	297713.34	-148697.24	588544.00

Table S14: Wald test results of all models.

Model	Variable	Chi-squared	df	p-value.
Model 1	(Intercept)	2589.38	1	0.00E+00
	SES quintiles	4775.81	4	0.00E+00
Model 2	(Intercept)	2733.59	1	0.00E+00
	SES quintiles	3912.53	4	0.00E+00
	Age (standardised)	11284.34	1	0.00E+00
	Sex (male)	0.74	1	3.91E-01
	Year of admission	450.86	8	2.42E-92
Model 3a	(Intercept)	2968.29	1	0.00E+00
	SES quintiles	2914.54	4	0.00E+00
	Age (standardised)	10709.63	1	0.00E+00
	Sex (male)	1.61	1	2.04E-01
	Year of admission	509.90	8	5.28E-105
	Respiratory insufficiency (present)	375.68	1	1.08E-83
	SES quintile × respiratory insufficiency	17.94	4	1.27E-03
Model 3b	(Intercept)	2887.27	1	0.00E+00
	SES quintiles	3497.17	4	0.00E+00
	Age (standardised)	10539.63	1	0.00E+00
	Sex (male)	0.58	1	4.47E-01
	Year of admission	483.73	8	2.18E-99
	Renal insufficiency (present)	433.68	1	2.58E-96
	SES quintile × renal insufficiency	38.11	4	1.06E-07
Model 3c	(Intercept)	2742.56	1	0.00E+00
	SES quintiles	4062.64	4	0.00E+00
	Age (standardised)	10922.17	1	0.00E+00
	Sex (male)	1.48	1	2.24E-01
	Year of admission	493.57	8	1.69E-101
	Cancer (present)	814.52	1	3.75E-179
	SES quintile × cancer	76.29	4	1.06E-15
Model 3d	(Intercept)	2717.69	1	0.00E+00

Model	Variable	Chi-squared	df	p-value.
	SES quintiles	3519.19	4	0.00E+00
	Age (standardised)	10865.78	1	0.00E+00
	Sex (male)	0.00	1	9.44E-01
	Year of admission	483.48	8	2.46E-99
	Cardiovascular insufficiency (present)	156.06	1	8.22E-36
	SES quintile × cardiovascular insufficiency	7.51	4	1.11E-01
Model 3e	(Intercept)	2762.30	1	0.00E+00
	SES quintiles	3034.13	4	0.00E+00
	Age (standardised)	11061.62	1	0.00E+00
	Sex (male)	0.33	1	5.65E-01
	Year of admission	446.68	8	1.91E-91
	Diabetes mellitus (present)	23.49	1	1.26E-06
	SES quintile × diabetes mellitus	5.52	4	2.38E-01
Model 3f	(Intercept)	2850.21	1	0.00E+00
	SES quintiles	3652.17	4	0.00E+00
	Age (standardised)	11617.52	1	0.00E+00
	Sex (male)	0.01	1	9.43E-01
	Year of admission	465.77	8	1.54E-95
	Cirrhosis (present)	505.02	1	7.70E-112
	SES quintile × cirrhosis	15.39	4	3.96E-03
Model 3g	(Intercept)	2899.83	1	0.00E+00
	SES quintiles	3897.67	4	0.00E+00
	Age (standardised)	11213.36	1	0.00E+00
	Sex (male)	3.58	1	5.84E-02
	Year of admission	437.52	8	1.75E-89
	Immunological insufficiency (present)	816.88	1	1.15E-179
	SES quintile × immunological insufficiency	102.37	4	3.07E-21
Model 4	(Intercept)	6241.72	1	0.00E+00
	SES quintiles	1671.77	4	0.00E+00
	Age (standardised)	8579.53	1	0.00E+00
	Sex (male)	1.42	1	2.33E-01
	Year of admission	566.52	8	3.67E-117
	APS (standardised)	73165.34	1	0.00E+00
	Respiratory insufficiency (present)	1634.66	1	0.00E+00
	Renal insufficiency (present)	0.05	1	8.27E-01
	Cancer (present)	1856.72	1	0.00E+00
	Cardiovascular insufficiency (present)	365.46	1	1.82E-81
	Diabetes mellitus (present)	95.34	1	1.61E-22
	Cirrhosis (present)	600.24	1	1.48E-132
	Immunological insufficiency (present)	675.55	1	6.20E-149

Table S15: Likelihood Ratio Test (LRT) results of the interaction terms of the interaction models (Model 3 a-g).

Interaction term	Log-likelihood (without interaction)	Log-likelihood (with interaction)	Chi-squared	Δdf	p-value
SES quintile × respiratory insufficiency	-204529.99	-204521.06	17.84	4	1.32E-03
SES quintile × renal insufficiency	-204434.19	-204415.49	37.41	4	1.48E-07
SES quintile × cancer	-203199.95	-203162.31	75.27	4	1.74E-15
SES quintile × cardiovascular insufficiency	-204870.81	-204867.08	7.46	4	1.13E-01
SES quintile × diabetes mellitus	-205183.21	-205180.46	5.51	4	2.39E-01
SES quintile × cirrhosis	-204149.65	-204142.00	15.29	4	4.14E-03
SES quintile × immunological insufficiency	-203452.58	-203401.75	101.68	4	4.33E-21

Table S16: Wald test results for the joint effect of SES stratified by comorbidity, based on the interaction models (Model 3 a-g).

comorbidity	Stratum	df	Chi-squared	p-value
Respiratory insufficiency	Absent	4	2914540	0.00E+00
Respiratory insufficiency	Present	4	617972	2.00E-127
Renal insufficiency	Absent	4	3497168	0.00E+00
Renal insufficiency	Present	4	203292	7.36E-37
Cancer	Absent	4	4062644	0.00E+00
Cancer	Present	4	197736	1.15E-35
Cardiovascular insufficiency	Absent	4	3519192	0.00E+00
Cardiovascular insufficiency	Present	4	323444	9.48E-63
Diabetes mellitus	Absent	4	3034136	0.00E+00
Diabetes mellitus	Present	4	787856	3.28E-163
Cirrhosis	Absent	4	3652168	0.00E+00
Cirrhosis	Present	4	56868	1.32E-05
Immunological insufficiency	Absent	4	3897672	0.00E+00
Immunological insufficiency	Present	4	210400	2.18E-39

Table S17: Sensitivity analysis of model 3 with the number of comorbidities instead of a specific comorbidity. SES quintile 3 and admission year 2019 were used as reference categories.

Variable	Odds Ratio (95% CI)	z-Statistic	p-value
(Intercept)	0.07 (0.07-0.08)	-56.57	0.00
SES quintile 1	1.67 (1.61-1.73)	27.34	0.00
SES quintile 2	1.09 (1.06-1.14)	4.83	0.00
SES quintile 4	0.89 (0.86-0.93)	-5.63	0.00
SES quintile 5	0.83 (0.79-0.86)	-9.27	0.00
Age (standardised)	1.67 (1.65-1.69)	95.65	0.00
Sex (male)	0.99 (0.97-1.01)	-1.32	0.19
Year of admission (2014)	0.98 (0.95-1.01)	-1.20	0.23
Year of admission (2015)	1.04 (1.00-1.07)	2.04	0.04
Year of admission (2016)	1.04 (1.00-1.07)	2.17	0.03
Year of admission (2017)	1.03 (1.00-1.07)	1.71	0.09
Year of admission (2018)	1.07 (1.03-1.10)	3.55	0.00
Year of admission (2020)	1.22 (1.17-1.26)	10.92	0.00
Year of admission (2021)	1.35 (1.30-1.40)	16.73	0.00
Year of admission (2022)	1.17 (1.13-1.21)	8.64	0.00
One comorbidity	1.44 (1.39-1.50)	17.85	0.00
Two comorbidities	2.37 (2.26-2.50)	33.41	0.00
More than two comorbidities	3.68 (3.39-3.98)	31.77	0.00
SES quintile 1 × one comorbidity	1.02 (0.96-1.07)	0.56	0.58
SES quintile 2 × one comorbidity	0.99 (0.94-1.05)	-0.38	0.71
SES quintile 4 × one comorbidity	1.06 (0.99-1.12)	1.79	0.07
SES quintile 5 × one comorbidity	1.11 (1.04-1.19)	3.29	0.00
SES quintile 1 × two comorbidities	0.90 (0.85-0.97)	-2.90	0.00
SES quintile 2 × two comorbidities	0.93 (0.86-0.99)	-2.17	0.03
SES quintile 4 × two comorbidities	1.19 (1.10-1.29)	4.44	0.00
SES quintile 5 × two comorbidities	1.36 (1.25-1.47)	7.39	0.00
SES quintile 1 × more than two comorbidities	0.80 (0.72-0.89)	-4.12	0.00
SES quintile 2 × more than two comorbidities	0.90 (0.81-1.01)	-1.82	0.07
SES quintile 4 × more than two comorbidities	1.18 (1.04-1.34)	2.53	0.01
SES quintile 5 × more than two comorbidities	1.29 (1.13-1.49)	3.64	0.00

Table S18: Extreme-case sensitivity analysis of model 4 where the minimum SES was imputed for patients that were not linked or were missing SES. SES quintile 3 and admission year 2019 were used as reference categories.

Variable	OR (95%CI)	z-statistic	p-value
(Intercept)	0.05 (0.04-0.05)	-78.92	0.00
SES quintile 1	1.48 (1.44-1.52)	27.44	0.00
SES quintile 2	1.15 (1.12-1.18)	9.88	0.00
SES quintile 4	0.94 (0.92-0.97)	-3.83	0.00
SES quintile 5	0.90 (0.87-0.93)	-6.26	0.00

Table S19: Extreme-case sensitivity analysis of model 4 where the maximum SES was imputed for patients that were not linked or were missing SES. SES quintile 3 and admission year 2019 were used as reference categories.

Variable	OR (95%CI)	z-statistic	p-value
(Intercept)	0.04 (0.04-0.05)	-79.13	0.00
SES quintile 1	1.54 (1.50-1.58)	30.71	0.00
SES quintile 2	1.04 (1.02-1.07)	3.09	0.00
SES quintile 4	0.95 (0.92-0.98)	-3.30	0.00
SES quintile 5	1.09 (1.06-1.13)	5.55	0.00

References

1. van de Klundert N, Holman R, Dongelmans DA, et al.: Data Resource Profile: the Dutch National Intensive Care Evaluation (NICE) Registry of Admissions to Adult Intensive Care Units. *Int J Epidemiol* 2015; 44:1850–1850h

2. NICE Data Dictionary [Internet]. *Sticht-Nice* [cited 2024 Sept 6] Available from: https://www.stichting-nice.nl/dd/#start

3. Brinkman S, de Jonge E, Abu-Hanna A, et al.: Mortality After Hospital Discharge in ICU Patients*. *Crit Care Med* 2013; 41:1229
